# Iron as the concert master in the pathogenic orchestra playing in sporadic Parkinson’s disease

**DOI:** 10.1007/s00702-021-02414-z

**Published:** 2021-10-12

**Authors:** P. Riederer, C. Monoranu, S. Strobel, T. Iordache, J. Sian-Hülsmann

**Affiliations:** 1 Clinic and Policlinic for Psychiatry, Psychosomatics and Psychotherapy, University Hospital Wuerzburg, University of Wuerzburg, Wuerzburg, Germany; 2 Department of Psychiatry, University of Southern Denmark, Odense, Denmark; 3 Institute of Pathology, Department of Neuropathology, University of Wuerzburg, Wuerzburg, Germany; 4 George Emil Palade University of Medicine, Pharmacy, Science and Technology of Targu Mures, Târgu Mureș, Romania; 5 Department of Medical Physiology, University of Nairobi, P.O. Box 30197, Nairobi, 00100 Kenya

**Keywords:** Iron in Parkinsonism, Parkinson’s disease, Iron transporter, Neuromelanin, Iron pathology, Neuroinflammation, Iron model, Ferroptosis, ɑ-Synuclein and iron, Virus–iron interaction, COVID-19, Hepcidin, SARS-CoV-2

## Abstract

About 60 years ago, the discovery of a deficiency of dopamine in the nigro-striatal system led to a variety of symptomatic therapeutic strategies to supplement dopamine and to substantially improve the quality of life of patients with Parkinson’s disease (PD). Since these seminal developments, neuropathological, neurochemical, molecular biological and genetic discoveries contributed to elucidate the pathology of PD. Oxidative stress, the consequences of reactive oxidative species, reduced antioxidative capacity including loss of glutathione, excitotoxicity, mitochondrial dysfunction, proteasomal dysfunction, apoptosis, lysosomal dysfunction, autophagy, suggested to be causal for ɑ-synuclein fibril formation and aggregation and contributing to neuroinflammation and neural cell death underlying this devastating disorder. However, there are no final conclusions about the triggered pathological mechanism(s) and the follow-up of pathological dysfunctions. Nevertheless, it is a fact, that iron, a major component of oxidative reactions, as well as neuromelanin, the major intraneuronal chelator of iron, undergo an age-dependent increase. And ageing is a major risk factor for PD. Iron is significantly increased in the substantia nigra pars compacta (SNpc) of PD. Reasons for this finding include disturbances in iron-related import and export mechanisms across the blood–brain barrier (BBB), localized opening of the BBB at the nigro-striatal tract including brain vessel pathology. Whether this pathology is of primary or secondary importance is not known. We assume that there is a better fit to the top-down hypotheses and pathogens entering the brain via the olfactory system, then to the bottom-up (gut-brain) hypothesis of PD pathology. Triggers for the bottom-up, the dual-hit and the top-down pathologies include chemicals, viruses and bacteria. If so, hepcidin, a regulator of iron absorption and its distribution into tissues, is suggested to play a major role in the pathogenesis of iron dyshomeostasis and risk for initiating and progressing ɑ-synuclein pathology. The role of glial components to the pathology of PD is still unknown. However, the dramatic loss of glutathione (GSH), which is mainly synthesized in glia, suggests dysfunction of this process, or GSH uptake into neurons. Loss of GSH and increase in SNpc iron concentration have been suggested to be early, may be even pre-symptomatic processes in the pathology of PD, despite the fact that they are progression factors. The role of glial ferritin isoforms has not been studied so far in detail in human post-mortem brain tissue and a close insight into their role in PD is called upon. In conclusion, “iron” is a major player in the pathology of PD. Selective chelation of excess iron at the site of the substantia nigra, where a dysfunction of the BBB is suggested, with peripherally acting iron chelators is suggested to contribute to the portfolio and therapeutic armamentarium of anti-Parkinson medications.

## Introduction

**Fig. 1:**
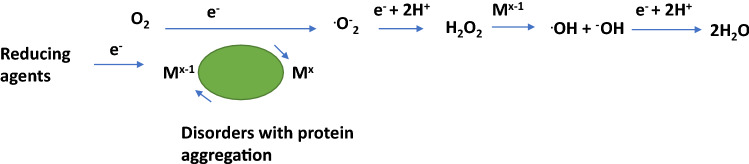
Generation of oxidative species by redox-active metals. M^x−1^ iron ions (eventually copper ions) in the reduced state, M^x^ iron/copper ions, O_2_ molecular oxygen, ^.^O_2_^−^ superoxide radical, H_2_O_2_ hydrogen peroxide, ^.^OH hydroxyl radical, ^−^OH hydroxyl ion

Evolution of the human being was and will never be possible without the action of a variety of metals, like sodium, potassium, calcium, zinc, copper, manganese, iron, aluminium, nickel and others. However, understanding their mode of action (MoA) was possible only by the development of highly sophisticated armamentaria of methodologies over the past decades. These methods allowed to get insights into the biological mechanisms of metal ions interaction with peptides/proteins, enzymes, nucleotides etc.. Transport, metabolism, metal deficit and accumulation, respectively, and metal toxicity became important research fields (Sigel et al. 2006) (Fig. 1).

In this review, we focus on “iron” and its role in the pathology of Parkinson’s disease (PD). The brain utilizes metal ions for many metabolic purposes. Compared with other tissues, the brain shows the highest metabolic rate and need for aerobic metabolism. Uptake of iron is not as ease, because the blood–brain barrier (BBB) is a natural barrier for iron-uptake. Therefore, iron is taken up using transport proteins, like transferrin. Transferrin binds iron and this complex after endocytosis releases iron intracellularly through divalent metal transporter 1. Iron exit from brain is controlled by ferroportin, hepcidin and ferroxidases (Vela 2018; Zecca et al. 2004b; Gerlach et al. 2021).

This condition is a vulnerable one for redox-active metals, like iron and copper. Indeed, reactive oxygen species (ROS) play a dominant role in the pathology of neurodegenerative disorders, like Parkinson’s disease (PD) and Alzheimer disease (AD). A majority of radicals and ROS arise from redox reactions of metals (Berg et al. 2004). Metals, like iron and copper in their reduced state reduce oxygen (O_2_) to superoxide^.^${\text{O}}^{ - }_{{2}}$ which is dismutated to hydrogen peroxide (H_2_O_2_). H_2_O_2_ crosses membranes and can be scavenged by glutathione peroxidase, catalase, etc. If not scavenged, H_2_O_2_ may react with reduced metals to generate hydroxyl radicals (^.^OH). As ^.^OH is an extremely reactive radical species, it reacts easily with lipids, proteins to form, e.g. protein aggregation products and even RNA and DNA adducts (Sigel et al. 2006). Indeed iron-associated disorders can be divided as (1) genetic disorders, like Friedreich’s ataxia (frataxin), neurodegenerations with brain iron accumulation (NBIA), pantothenate kinase 2-associated neurodegenerations, neuroferritinopathy (mutation of ferritin light chain) and aceruloplasminemia (together with copper; mutations in the ceruloplasmin gene), hemochromatosis (mutations in the HFE gene) and (2) disorders with protein aggregation, like PD (and eventually associated with copper pathology) (Schneider 2016; Gerlach et al. 2006). However, the notion is important, that iron accumulation can be detected in many other neurodegenerative disorders, like AD, Huntington’s chorea, multiple system atrophy (MSA), progressive supranuclear palsy (PSP). In all these disorders iron accumulation is specifically located in brain regions associated with the key pathology of the disorder. For example, in AD increased iron concentration has been found in cortical brain areas (Ward and Crichton 2019; Seitelberger 1964), while there is iron pathology in PD detected in the substantia nigra pars compacta (SNpc); in MSA not only there is an accumulation in the SN but also in the striatum and the same holds true for PSP. From such data, it is not farfetched to assume that iron pathology may be a secondary mechanism facilitating neurodegeneration rather than a primary causal trigger. To support this assumption, it has been demonstrated, that iron shows an age-dependent increase and distribution in the various brain areas, as already shown by Spatz (1922), Müller (1922), Hallgren and Sourander (1958), Schmidt (1940), Volkl and Ule (1972), and Ule et al. (1974).

Early measurements of total iron in qualitative histological iron stainings (Schmidt 1940; Müller 1922; Spatz 1922) and in quantitative analyses have been reported in healthy individuals in many brain regions with the highest concentrations in globus pallidus, SN, putamen, caudate nucleus and red nucleus (Hallgren and Sourander 1958; Ule et al. 1974; Volkl and Ule 1972; Stern et al. 1967). This is of interest, because there are no regional differences in the concentrations of zinc, calcium and magnesium (Ule et al. 1974).

Of interest is the notion, that non-heme iron can be detected especially in mitochondria and microsomes (46% on average). 14% were found in nuclei and the remaining 40% in the soluble fraction (Hallgren and Sourander 1958). Ule et al. (1974) assumed that iron, copper and zinc are bound to functional active macromolecules, like coenzymes, structural proteins or deposited pigments, although not much detailed, Spatz already mentioned in 1922 (on page 312) (Spatz 1922), that there is iron bound to pigment in the SN.

### Iron in Parkinson’s disease

Lhermitte et al. (1924) have described abnormal deposits of iron in globus pallidus but not in the SN of a patients with akinetic-rigid PD. Using different technologies, like X-ray fluorescent spectroscopy (Earle 1968), magnetic resonance imaging (Rossi et al. 2013; Lee and Lee 2019; Drayer et al. 1986), inductively coupled plasma spectroscopy (Dexter et al. 1987), X-ray microanalysis (Hirsch et al. 1991; Kienzl et al. 1995; Jellinger et al. 1992), spectrophotometry (Sofic et al. 1988), T2*-, R2*- susceptibility weighting imaging (Wang et al. 2016; Graham et al. 2000), transcranial sonography (Becker et al. 1995; Berg et al. 2005), quantitative susceptibility mapping (Pyatigorskaya et al. 2020), FDRI -method, iron relaxometry techniques, PRISME MRI and other imaging methods an increase of total iron in the SNpc could be established, as summarized by Chen et al. (2019), Genoud et al. (2020), Gerlach et al. (1994, 2006, 2021) and Feraco et al. (2021). In patients with von Economo encephalitis epidemica at late stages of the disease an accumulation of iron in the SN has been mentioned by Seitelberger (1964). In contrast to previous observers, Rutledge et al. (1987) did not find that MR signal correlated between Perls’ stain and the signal void exists everywhere. This early finding has been noted also by other researchers and has been discussed recently in more detail. Haacke et al. (2005) examined the response of the magnetic resonance visible iron in tissue that produces signal changes in both magnitude and phase imaging and assumed that these images seem to correlate with brain iron content, perhaps ferritin specifically.

The mismatch of T2*-weighted MRI and Perl stains has been noted also by Blazejewska et al. (2013) in an imaging study of 2 subjects with unknown neurological condition (67 resp. 46 years old) and one patient diagnosed with PD (75 years old). Brains were fixed in 10% formalin (see our comments below). Although the data from this study are only preliminary (number of individuals, no healthy controls for comparison, age differences, 10% formalin for fixation), they relate the problem of the mismatch to a higher concentration of iron (II) in PD brain due to the fixation process and/or increased oxidative stress, while Perl stains only iron (III). Certainly measurements of the proportion of divalent/trivalent iron (Sofic et al. 1988; Galazka-Friedman et al. 1996) are a challenge due to methodological uncertainties (Friedman and Galazka-Friedman 2012; Hare et al. 2012).

The review of Haacke et al. (2005) seems to be a good basis for the development of image analyses to improve the detection of iron with T1 and T2 techniques. In fact, Haacke’s group (Mittal et al. 2009) described susceptibility weighted imaging (SWI), which is 3–6 times more sensitive than conventional T2* weighted gradient echo sequences and gave example for clinical applications. The review of Feraco et al. (2021) describes the development of iron imaging techniques with details for nigrosome imaging, neuromelanin-sensitive sequences, iron-sensitive sequences and advanced diffusion weighted imaging techniques, which all afford new insights into the non-invasive study of the SNpc.

An interesting study using T1 and T2* mapping in iron-overload-related heart failure may give an answer for preferring T1 or T2* mapping when measuring iron concentrations (Torlasco et al. 2018). These authors concluded their studies as follows: T1 and T2* are concordant as long as the slopes for T2* are high. Nevertheless, while the sensitivity of conventional MRI sequences, i.e. T2 or T1 weighted, has been considered as poor for the detection of early PD (see Feraco et al. (2021)) these authors state, that quantitative susceptibility mapping and R2* may be effective tools for early detection as well as for the dynamic progression of PD.

For the detection of free (labile) iron, it should be critically noted that there are several caveats to be considered (Hare et al. 2012; Friedman and Galazka-Friedman 2012), like a metal transfer due to sampling, fixation and storage of post-mortem brain tissue, impact of analytical sensitivity (Hare et al. 2012), hemispheric asymmetry with a higher concentration of iron in the left hemisphere (Xu et al. 2008) and most importantly the measurement of changes in total iron and the labile iron pool (Hare et al. 2012). Here we focus on some methodological problems which have direct influence for the interpretation of findings related to free (labile) vs. total iron and their correlation to the pathology of PD: (1) as total iron is increased, does it mean, that free (labile) iron is increased too and is the relation of free to bound (ferritin, neuromelanin (NM)) iron an aspect to be considered important for the pathology of PD? and (2) is the increase of iron correlated to the staging of PD progression and is there specificity of increased SN iron in PD?

*Ad (1)* Free vs. bound iron: although an increase of free (labile) iron has been published by Wypijewska et al. (2010), there are no other publications supporting these data. This fact is not surprising, because quantitative detection of free cytoplasmic iron in formalin-fixed tissues is not possible due to the destruction of tissue membrane substructures. If not formalin-fixed, fresh tissue can be used or fresh-frozen tissue. In the latter case, however, freezing protocols would have been to be used extremely carefully avoiding shock-freezing protocols and using instead slow/mild temperature reducing protocols (Hare et al. 2012). To detect free iron in fresh tissue or in frozen SN (frozen down using membrane protecting protocols) such tissue would have been to be merged using extremely tight methodology, e.g. squashing of tissue, but not using cutting technologies under cooling conditions. All these analytical procedures are extremely tricky and would have been followed by membrane filtration using ultra capsules to separate free from bound iron with the time-dependent possibility that the equilibrium of free and bound iron is changed. To avoid such complicated methodology tissue in toto is placed into fluid*;* then free tissue iron is assumed to diffuse into such fluid with the possibility to be easily measured. However, iron equilibrium of free and bound iron might be changed as this is a time consuming strategy and longer time offers a greater possibility for changing this equilibrium in favour of free iron concentration.

Ad(2) iron and staging of PD

Although, and mentioned above, there is overwhelming evidence that iron is increased in the SN of sporadic PD, nevertheless, there are a few reports from post-mortem analyses showing that iron is not changed when compared to controls (Gałazka-Friedman et al. 1996; Friedman et al. 2009; Uitti et al. 1989; Ryvlin et al. 1995). Reasons for these discrepancies are several fold and include (1) fixing protocols of post-mortem tissue, e.g. has the whole brain or just one of the hemispheres been fixed in formalin? If so, which one? Were brain slices or special regions fixed as well? Furthermore, knowledge about the concentration of formalin, number of repeated exchanges (N) of formalin, times between these repeated fluid exchanges, duration of formalin fixation, use of phosphate-buffered formalin or formalin fixation without buffered fluid, washout phases of formalin (N) with water resp. considering also the composition of this fluid would be of interest. This is, because and as mentioned already by Spatz et al. (1922), who described the loss of iron in long-term treated brains with formalin. In addition, such information would give evidence for the redox potential of fluids integrated into the fixation protocol. Our own experience shows the phosphate-buffered formalin is superior to pure formalin for histological/immunohistological research strategies (Heinsen, pers.comm.); (2) length of tissue storage and storage conditions, e.g. storage in formalin or as paraffin-embedded blocks (see also Hare et al. 2012; Friedman and Galazka-Friedman 2012; Blazejewska et al. 2013). Furthermore, time between death and brain autopsy as well as between brain autopsy and fixation protocol, temperature condition, dissection uncertainties of the substructure of the SN, e.g. SN pars compacta. SN pars reticulata (Sofic et al. 1991) or SN to red nucleus, have been suggested to influence measurements of iron in post-mortem brain tissue (Friedman and Galazka-Friedman 2012; Hare et al. 2012; Blazejewska et al. 2015). In addition, it would be of great importance to learn about the origin of post-mortem tissue. For example, have post-mortem brain tissue of controls and PD collected at the same neuropathological institute or have they been collected at different neuropathologies? (Earle 1968; Schrag et al. 2010; Gellein et al. 2008).

While such details are mostly not reported in respective publications, the notion may be of interest namely that Spatz (1922) reported the disappearance of iron staining in long-term formalin fixed tissue. Another aspect is the fact that there are different progression rates of PD, which may indeed influence iron-induced pathology. Indeed, no increase of iron concentration has been found in incidental Lewy body disease (ILBD) (Dexter et al. 1991) and in PD with milder SN pathology (Bartzokis et al. 1999; Riederer et al. 1989)). Bartzokis et al. (1999) studied iron content in the extrapyramidal system in early- and late-onset PD by MRI-technique. This group showed that there were significant increases in early-onset PD in field-dependent R2 increases (FDRI) in the SN, putamen and globus pallidus, while later onset PD subjects had significantly decreased FDRI in the SN pars reticulata. These authors concluded that the increase of iron in the SN of PD as measured post-mortem is in line with the decreased FDRI measure in late-onset patients detected in their study (Bartzokis et al. 1999). Iron accumulation was not significant in different regions of interest in newly diagnosed patients with PD after adjustment for age (Dashtipour et al. 2015). However, these authors calculated an average phase value from the left and right hemispheral side, thus not considering different pathological states in the regions of interest, like the SN. Indeed, post-mortem findings showing an increase of SN iron and performed in tissue from advanced PD are overwhelming as mentioned above. However, a conclusion, that increase of total iron is correlated to advanced PD stages is questionable considering SN-iron-related imaging, which shows increased iron-related alterations in eventually pre-symptomatic individuals as published by Becker et al. (1995) and Berg et al. (1999). However, later clinical studies showed, that hyperechogenicity detected by transcranial ultrasound imaging is a highly increased risk for PD in elderly individuals (Berg et al. 2011) only, while other studies demonstrated SN hyperechogenicity also in other disease entities (Berg 2011). With MRI (3 Tesla magnet) using a multiple gradient echo sequence designed for rapid single-scan mapping of the proton transverse relaxation rate R2* Martin et al. (2008) demonstrated increased iron content in early PD. Hyperechogenicity of the SN has been shown in the 6-hydroxydopamine model of PD to depend on iron accumulation and microglia activation (Zhu et al. 2017). Such studies are important in order to get insights into the role of iron as a trigger of PD pathology and/or a deleterious disease progression factor. As the SN is the predominant brain region showing increased iron deposits in PD, the question raises as to the reasons of such localized pathology. Animal studies give evidence for a disturbed BBB in 6-hydroxydopamine lesioned animals with increase of SN iron and albumin (as indicator of a leaky BBB) (Oestreicher et al. 1994; Arendash et al. 1993). Alternatively, it has been suggested, there might be disturbances in the mechanisms of iron-uptake, iron-transport and iron-storage.

### Iron-induced pathology in the SN pars compacta of PD

**Fig. 2:**
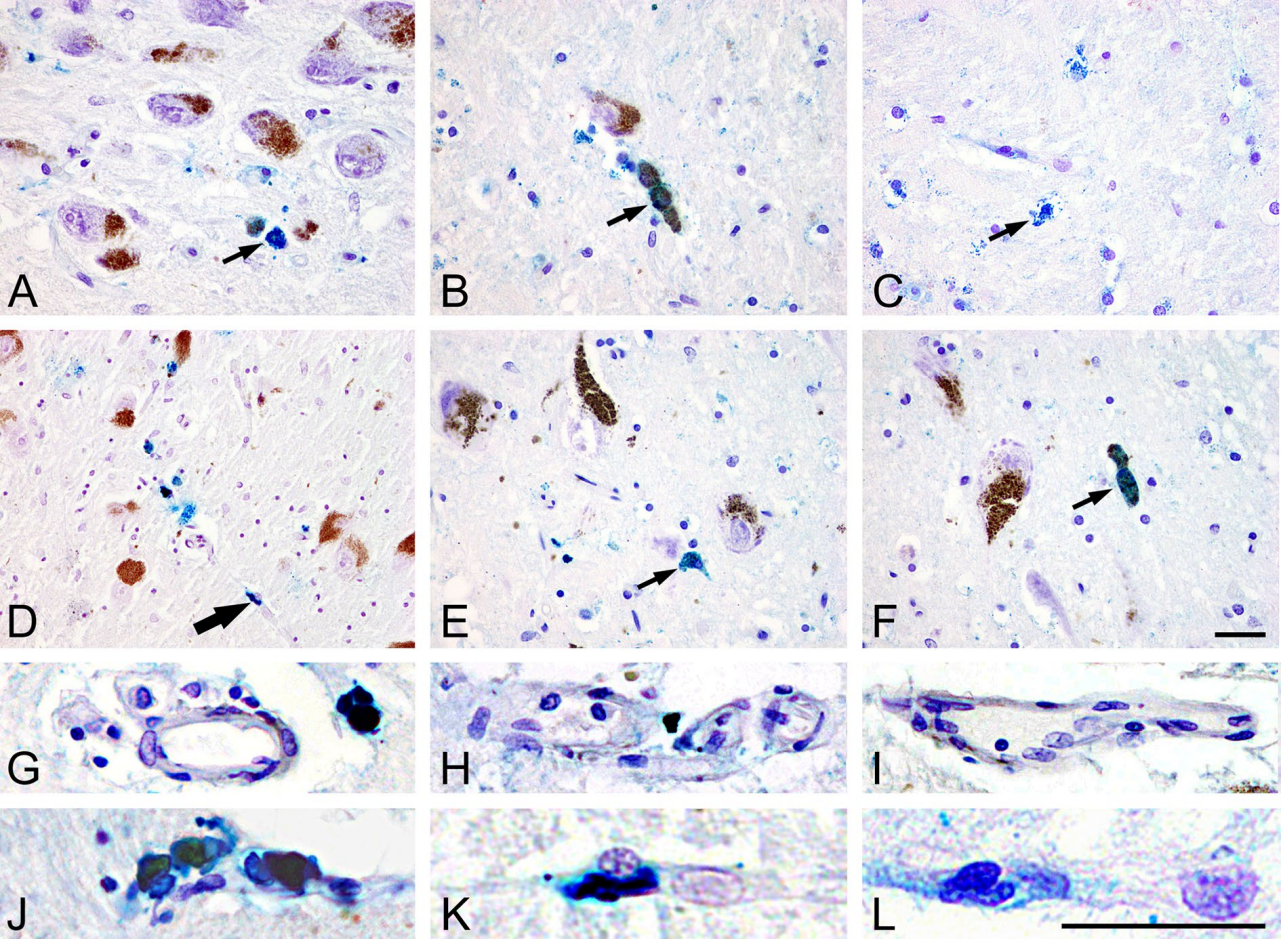
Detection of iron deposits (Fe III) using Berlin blue reaction: intracytoplasmic fine granules in neuromelanin containing neurons of SN both in controls (**A**, **B**) and in PD cases (**D**–**F**) as well in glial cells in controls (**C**) (thin arrows). Coarsely deposits in capillary walls in PD cases (e.g. **D**) (thick arrow), whereas in controls (**A**–**C**) such reaction is missing (200× magnification, scale bar 50 µm). *G-I* Iron-free vessels in controls, *J-L* iron deposits in vessels of PD brains

Transferrin has been shown to be decreased in the SN of PD by 35% (Ayton et al. 2016). Such data agree with those by Morris et al. (1994), but is correlated with the loss of nigral neurons (Morris et al. 1994). Loeffler et al. (1995) concluded from their studies, that the transferrin/iron ratio, a measure of iron mobilization capacity, provides evidence for a disturbance in iron metabolism in PD (Loeffler et al. 1995). However, lactoferrin has been shown to be increased in PD (Faucheux et al. 1995; Leveugle et al. 1996). There is also evidence that the expression of iron export protein ferroportin is increased in the SN of PD, while the iron-storage protein ferritin expression is unchanged (Visanji et al. 2013), increased (Riederer et al. 1989) or decreased (Dexter et al. 1991). As all this information is not too convincing for the proof, that a disturbance of iron transport is causally responsible for triggering an increased brain and especially SN accumulation of iron, the increase of iron in the SN of PD might be secondary to a disturbed BBB as demonstrated in animal models of PD (Oestreicher et al. 1994; Arendash et al. 1993). Increased staining of SN capillaries in post-mortem SN as shown in Fig. 2 would be in line with such assumptions and data presented by Faucheux et al. (1999). “Therefore, the driving force of triggering PD might be the continuous uptake of free iron through a disturbed BBB at the site of the substantia nigra (SN). Intraneuronal facilitation of oxidative stress (OS), driven by iron, may disturb mitochondrial respiratory chain activity and may contribute to the generation of fibril ɑ-synuclein (ɑ-syn) and reduction in proteasomal activity. NM-induced toxicity, protein aggregation and generation of Lewy bodies (LB) are the consequence. In addition, glial activation will release several compounds, which enhance and synergistically drive toxic cell death cascades” (Riederer 2004). Evidence for a disturbed BBB comes from studies by Faucheux et al. (1999) and experimental studies using 6-hydroxydopamine, showing an increased uptake of iron at the site of the SN (Oestreicher et al. 1994). In addition, binding parameters of the iron-transporting protein transferrin are changed in PD (Gerlach et al. 2006) and an augmented expression of the divalent cation transporter has been shown in SN neurons (Qian and Wang 1998). Also using a unilaterally MPTP-treated monkey model increased iron was found in degenerating dopamine cells, glia and neuron surrounding matrix (Temlett et al. 1994).

**Fig. 3:**
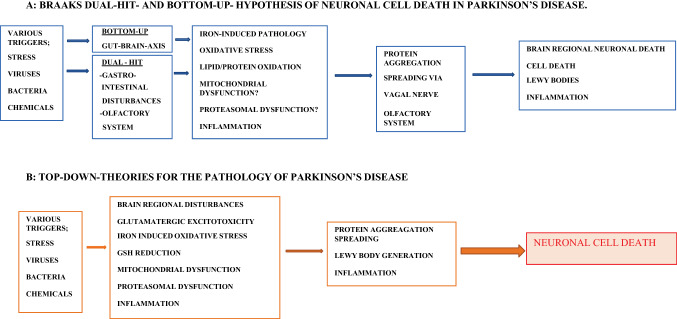
Pathological events triggering Parkinson’s disease

Conclusions from this part are

- IRON increases with age.
- IRON transfer through BBB is increased.
- IRON export from brain is increased.
- IRON is increased in SN blood vessels.
- IRON storage by ferritin and neuromelanin is dysregulated.
- Free/labile IRON increases and induces Fenton reaction leading to toxic hydroxyl radicals.
- Ferroptosis causes proteasomal defect, mitochondrial disturbances and cellular cytotoxic processes, lysosomal defects, apoptosis and autophagy dysregulation, all together causing cell degeneration. Ferroptosis is characterized by an accumulation of lipid hydroperoxides (Dixon Scott et al. 2012). The sensitivity to ferroptosis is linked to numerous biological processes and in particular to iron, glutathione, phospholipid, NADPH, coenzyme Q_10_ and polyunsaturated fatty acid metabolism (Yan and Zhang 2020; Stockwell et al. 2017). A deficient regulation of ferroptosis has been found in MPTP-treated mice (Tapias 2019), a model of PD pathology (Fig. 3).

This scenario seems to be evident for a pathological mechanism in which iron is involved in the progression of PD. However, it cannot be excluded that iron is also a key for triggering PD in the gut/olfactory system as is suggested by the interaction of the type-II transmembrane serine protease TMPRSS6/hepcidin interaction.

### Iron related animal models of PD

The assumption, that iron plays a predominant role in the pathogenesis of dopaminergic cell death in PD has favoured experimental studies to further elucidate pathological mechanisms underlying iron-induced pathology (Sengstock et al. 1992; Wesemann et al. 1994).

As described in detail by Gerlach et al. (2021), the unilateral intranigral injection of ferric iron at a low μg dose causes time-dependent altered motor behaviour in rats, which is accompanied by an average 95% reduction of the dopamine concentration in the ipsilateral striatum and a smaller reduction of its metabolites 3,4-dihydroxyphenylacetic acid and homovanillic acid. This is accompanied by reactive gliosis, iron-stained astrocytes and activated microglia already early after iron SNpc intoxication (Gerlach et al. 2021). To further elucidate the potential of an NM–iron-induced neurotoxicity, a rat model based on the intranigral injections of human NM-bound ferric iron into the SN has been developed. Injection of a NM-ferric iron suspension (0.139 μg iron) into the ventrolateral region of the left SNpc leads to 50% reduction of dopamine neuron number eight weeks after injection (Double et al. 2003b; Gerlach et al. 2021). At this dose of iron, no motor abnormalities could be observed and there was no reduction of dopamine suggesting subclinical dopaminergic lesions under these experimental conditions (Gerlach et al. 2021). Multiple animal studies and experimental work using cell culture systems as described in more detail by Gerlach et al. (2021) confirm that iron and the NM–iron complex are important risk factors for the proper functioning of dopaminergic neurons in the SNpc. However, the notion is of interest, that in PD a significant correlation between dopamine concentration and iron content is relevant only in the putamen but not in the SN (Gerlach et al. 1994). Under physiological conditions the putamen shows high concentrations of dopamine but only rather low levels of iron. This finding is vice versa in the SN. Therefore, one may assume that neither dopamine nor iron alone are primary toxins for the degenerating process related to the putamen and the SN.

As it has been described in more detail (Sian-Hulsmann and Riederer 2021), several hypothesis have been created to elucidate the pathological pathway(s) leading to PD. Braak’s “bottom-up hypothesis” (Braak et al. 2003b) claiming, that the pathology spreads in a prion-like manner from the gut via the vagus nerve and/or the olfactory system to brain regions (dual-hit-hypothesis), including the SNpc, has been challenged by Jellinger and others (Jellinger 2019), since only about 50% PD patients can be related to this pathology. Therefore, other hypotheses have been created to at least fill the gap and to provide evidence for other pathological pathways. One of these more recent hypotheses is the “top-down-hypothesis”, which postulates a cortical–striatal excitation stress, finally leading to a striato-nigral retrograde process, that includes ɑ-syn pathology (Foffani and Obeso 2018; Urban et al. 2020). Support for this are neuropathological findings showing that early in PD only 30% of dopaminergic neurons of the SNpc are degenerated but about 60% of its dendrites (Cheng et al. 2010). A third causal pathology, the “threshold hypothesis”, has been forwarded by Engelender and Isacson (Engelender and Isacson 2017), which collectively summarizes a variety of vulnerability factors.

In this respect, it is noteworthy, that already Lhermitte et al. (1924) reported an increase of iron in the globus pallidus (but not in the SN) in a patient with PD. This has been confirmed by Dexter et al. (1991) and Griffith et al. (1999) suggesting a possible retrograde pathological process. Indeed, multiple pathological processes and toxin interactions are suggested to be responsible for triggering and processing PD (Hare and Double 2016; Riederer et al. 2019; Sian-Hulsmann et al. 2011). In particular, evidence accumulates, that the risk for triggering pathological processes increases, when there is a high redox load (Berg et al. 2004), e.g. resulting from rather high iron concentration, tyrosine hydroxylase activity and dopamine concentrations, as in the SN (Riederer et al. 1992; Sofic et al. 1992; Gerlach et al. 1994).

### Tyrosine hydroxylase–iron relationship

Rausch et al. (1988) reported on the activity of tyrosine hydroxylase (TH) in post-mortem brain tissue of controls and PD under stimulating conditions in the presence of iron (II) and phosphorylating agents, like cAMP, exogenous protein kinase, calcium plus calmodulin and ATP. TH in control tissue was stimulated by 1 mM iron (II) by 13-fold. Although the activity of TH in striata of PD was 60% of controls, stimulation with 1 mM iron (II) reached an 11-fold increase of TH activity. This finding was similar to that of controls as was the Km-value of TH in controls and PD (Rausch et al. 1988; Riederer et al. 1988). Soluble TH interacts with ɑ-syn and an increase of ɑ-syn leads to reduction of TH activity. Therefore, it is hypothesized that iron-induced enhancement of TH activity by reducing ɑ-syn activity increases dopamine concentration. Furthermore, reaction of dopamine with iron causes ɑ-syn aggregation (Sian-Hulsmann et al. 2015).

### Neuromelanin-associated iron toxicity

NM is an insoluble complex organic polymer, which is accumulated in neuromelanin granules of various brain regions and most important in the SN and the locus coeruleus, brain regions, which are predominantly involved in the pathogenesis of PD (Marsden 1983). In fact, Hirsch et al. (1988, 1989) has shown, that it is the NM-containing dopaminergic neurons of the SN, which are primarily degenerating in PD. As such, NM received much interest to elucidate the role of NM as pathological component of the degenerating process. Here of special importance is NM capacity to bind metals and in particular iron (Gerlach et al. 2021; Youdim et al. 1989). This organic polymer binds iron with a high- and low-affinity binding characteristic (Ben-Shachar et al. 1991) and NM has been shown to be the major iron-storing structure in neurons while ferritin is located especially in the glia. Of notice is, that intraneuronal iron homeostasis is guaranteed by NM (Ben-Shachar et al. 1991; Youdim et al. 1990, 1989). As determined by Gerlach et al. (1995) in isolated NM from post-mortem SN by Mössbauer spectroscopy iron bound to NM consists exclusively of ferric iron, that is bound to ferritin-like oxyhydroxide cluster (Gerlach et al. 1995, 2021). The notion is of importance, that in dopaminergic neurons iron-mediated oxidation of dopamine might be responsible for the generation of NM (Double et al. 2000; Gerlach et al. 2008; Zecca et al. 2004a; Riederer et al. 2019).

Indeed, there is a long tradition to discuss the question, whether biosynthesis of NM is a spontaneous chemical out-oxidation reaction of dopamine/noradrenaline or whether some, but not all TH positive dopaminergic/noradrenergic neurons of the SN/locus coeruleus generate NM via a genetic programme (Zecca et al. 2001; Sulzer et al. 2000; Greggio et al. 2005; Ikemoto et al. 1998; Tribl et al. 2007; Carballo-Carbajal et al. 2019; Vila et al. 2019; Bellinger et al. 2011; Xu et al. 1997; Plum et al. 2016). This aspect is challenged by the fact, that (a) not all TH containing dopaminergic neurons generate NM as intraneuronal component (Hirsch et al. 1988, 1989) and (b) that treatment of PD patients with L-DOPA does not increase NM in remaining dopaminergic neurons (Jellinger and Paulus 1992), although it increases dopamine in remaining dopaminergic neurons. This is supported also by experimental studies showing that chronic levodopa is not further aggravating toxicity in rats with a nigrostriatal lesion (Murer et al. 1998). Furthermore, tyrosinase has been shown to be expressed in a subpopulation of dopaminergic neurons (Greggio et al. 2005; Carballo-Carbajal et al. 2019; Zecca et al. 2001; Miranda et al. 1984; Xu et al. 1997; Tief et al. 1998; Higashi et al. 2000) as summarized by Zecca et al. (2001). Tyrosinase has not always been detected in human SN (Ikemoto et al. 1998; Tribl et al. 2007) probably due to low sensitivity of methods used (Carballo-Carbajal et al. 2019). Even if the enzymatic expression seems to be extremely low, this does not exclude the possibility that tyrosinase synthesizes NM in an age-related manner in a subpopulation of dopaminergic neurons in the SN. Biochemical studies unravelling the kinetics of human tyrosinase may shed lights on the characteristics underlying NM biosynthesis (see e.g. Young et al. (2020). Besides tyrosinase, there might be other enzymes able to generate NM. As peroxidase is present in lysosomes, as well as NM, it has been suggested that this enzyme forms NM (Okun 1997). Indeed, peroxidase has been shown to be increased in post-mortem PD brain (De Iuliis et al. 2002). Also, by using subcellular proteomics, glutathione peroxidase 4 (GPX4) has been shown to be colocalized with NM in SN neurons (Tribl et al. 2005). Immunohistochemistry shows that GPX4 is up-regulated in neurons of the SN and associated with dystrophic axons in the striatum of PD (Bellinger et al. 2011) suggesting, that GPX4 colocalizes with alpha-synuclein (α-syn) positive nigral LB and dystrophic TH-positive fibres in the putamen. As glutathione (GSH) is reduced in PD (Dexter et al. 1991; Riederer et al. 1985) and even in ILBD (Dexter et al. 1994) this might indicate a response to OS leading to enhanced NM pathology. Nevertheless, NM protects neurons from excess free iron by its high iron-binding capacity and at least this mechanism operates until saturation of NM is evident (Ben-Shachar et al. 1991; Gerlach et al. 2003; Double 2006; Mochizuki et al. 2020; Mochizuki 1993). Thereafter and by unknown reasons iron may be released and promotes harmful redox reactions (Berg et al. 2004). Here Fenton reaction induced OS has been suggested a major pathological process including reaction with proteins like α-syn (Riederer et al. 2019; Shamoto-Nagai et al. 2006; Zucca et al. 2017). The toxicity underlying degenerating processes in the SN of PD seem to include components like α-syn, NM, iron as well as dopamine/dopamine oxidation products (Mandel et al. 2004; Riederer et al. 2019). NM increases with ageing (Zucca et al. 2018; Mann and Yates 1983; Vila et al. 2019) and NM deposition is associated with α-syn accumulation in aging neurons (Xuan et al. 2011). As in PD there is a greater overall reduction in the amount of melanin within remaining cells (15% in SN, 25% in locus coeruleus) because of a more severe (80%) loss of heavier pigmented cells, this further contributes to assume a massive disturbance of the iron–NM interaction with the consequence, that iron is set free from NM (Fasano et al. 2006). NM-sensitive imaging displayed reduced NM levels in the ventral (− 30 ± 28%) and dorsal tiers (− 21 ± 24%) as compared to controls (Martin-Bastida et al. 2017), thus agreeing with imaging studies performed by Pavese and Tai (2018), Hansen et al. (2016), Zupan et al. (2019).

As iron also undergoes an increase during ageing (Zecca et al. 2004a) the risk for a disturbance of iron homeostasis enhances and this may explain the vulnerability of dopaminergic neurons for pathological processes including disruption of endosomal and lysosomal function via multiple mechanisms as described in more detail by Perrett et al. (2015), Tribl et al. (2006), Plum et al. (2016), Isaias et al. (2016), Burbulla and Krainc (2019), Zecca et al. (2008), Carballo-Carbajal et al. (2019), Riederer et al. (2019), Pan et al. (2012). Support for this comes from studies showing both structural changes of NM (Double et al. 2003a) associated with a loss of iron binding to NM in PD (Fasano et al. 2006). Experimental studies using unilateral intranigral injections of ferric iron as well as unilateral injections of human NM-bound ferric iron into the SN of rats are in line with the suggestion, that the toxic couple NM–iron plays a predominant role in the pathogenesis of SN related dysfunction and degeneration in PD (Gerlach et al. 2021). In addition, experimental studies in MPTP-lesioned hemi-parkinsonian African Green monkeys show contralateral hemi-parkinsonism with significantly elevated iron compared to the unlesioned side (Temlett et al. 1994). In a seminal publication of Kurt Jellinger, he and his co-workers studied human post-mortem SNpc by using a X-ray-microanalysis technique. They found “weak but significant iron peaks—similar to those of synthetic melanin-iron (III) complex—only in intraneuronal highly electron-dense NM-granules of SNpc cells of PD plus AD. No detectable iron was seen in non-melanized cytoplasm of SNpc neurons and in the adjacent neuropil in PD and controls, in LBs in SNpc neurons of PD” (Jellinger et al. 1992). These findings demonstrated a NM–iron complex in dopaminergic SNpc neurons in PD supporting the idea, that a NM–iron interaction contributes significantly to dopaminergic degeneration in PD and AD (Jellinger et al. 1992; Kienzl et al. 1995). This data in PD has been confirmed by Good et al. (1992) and also recently via magnetic resonance transverse relaxation times (T_2_ and T_2_*) studies in the SNpc (Lee et al. 2020). An increase in iron levels in the SN of PD by post-mortem R_2_* or SWI measurements were observed also by Wang et al. (2016). Mean R_2_* in the SNpc defined by NM-sensitive MRI is significantly increased in PD (Martin-Bastida et al. 2017; Priovoulos et al. 2020; Langley et al. 2019; Huddleston et al. 2019). There is general agreement that NM is an excellent chelator of trace elements and especially iron. Therefore, and under physiological conditions NM protects neurons from toxic compounds (Fink et al. 2019; Zucca et al. 2018). In the case of increased iron support, NM may render saturated by iron. If so, excessive iron cannot be bound to NM and may cause generation of free hydroxyl radicals in the cytoplasm, OS with peroxidation of lipids, proteins, like α-syn, etc. followed by neurotoxicity, degenerating processes and neuronal loss. Here the notion is of interest, that there is a covalent linkage between α-syn and NM from the early stages of the disease on (Halliday et al. 2005; Fasano et al. 2006). More recent findings from a proteomic characterization of human post-mortem NM granules indicate, that about 1,000 proteins are bound and have been identified (Plum et al. 2016). A major question then is, what is the physiological function of TH-positive NM-containing vs. TH positive NM-negative dopaminergic neurons?

### General aspects of Parkinson pathology

PD represents a progressive neurodegenerative disease. It mainly afflicts the older population, although juvenile cases have been reported. The principal pathology of the disorder is characterised by the depletion in striatal dopamine content as a direct consequence of the degeneration of the nigro-striatal tract. The dopamine reduction largely contributes to many of the motor features exhibited by the disease.

There are other neuropathological features such as the presence of intracellular inclusions, LB in the SN and other afflicted areas in the brain. Although LB are not exclusive to PD and have been found in other diseases, nevertheless, its presence in the SN is paramount for the diagnosis of the illness. Indeed, LB diseases have been considered a spectrum including incidental LB disease, idiopathic PD, dementia with LB and Alzheimer’s disease (Jellinger and Korczyn 2018). Interestingly, the prevalence of these structures appears to increase with age from 3.8 to 12.8% between the sixth and ninth decade (Gibb and Lees 1988b).This concords with the notion that increasing age is predisposing factor in the manifestation of the disorder. Interestingly studies suggest that although there is similar LB pathology in young onset (less than 45 years) and older onset (over 70 years); however, the younger onset group exhibited a greater (24%) loss in the SN. However, there appears to be some underlying pre-requisite which determines the development of the neurodegenerative disorder. A genetic predisposition may confer vulnerability of the nigral neurons to the onslaught of the mechanisms operational in the illness (Lewis and Cookson 2012).

Increasing evidence supports the involvement of an imbalance or dysfunction of the cellular protein homeostasis as an important factor in the pathogenesis of PD and other related disorders. Indeed, LB comprise mainly of misfolded proteins such as α-syn but iron staining can be seen occasionally in the halo of LB (Jellinger et al. 1990). The appearance of these α-syn/LB inclusion clearly reflects an inability of the proteasome to degrade these unwanted inclusions and an indisposition of the protein homeostasis system. Subsequently, the cells with α-syn affect normal healthy neurons and spread in a prion-like manner, the “host to graft transmission” hypothesis (Recasens et al. 2014).

α-syn may exert its destruction via iron mode of cellular toxicity. Indeed, this concept is supported by its ability to function as ferrireductase (Sian-Hulsmann and Riederer 2020). Iron-dependent ferrireductase is involved in iron metabolism and possibly under pathological conditions, it is possible that in the pathological state it might not function at its full capacity and thus build-up total nigral iron in PD. Studies using retinal cells suggest that α-syn aggregates, interferes with iron autophagy and blocks its release from ferritin (Baksi and Singh 2017). Interestingly, unusual iron-responsive elements have been reported to be present in the 5’-untranslated region of α-syn (Ma et al. 2021), which may contribute to its oligomerisation and aggregation in the more advanced stages of the illness. Indeed, the absence of changes in iron levels in incidental Lewy body disease (Dexter et al. 1994), suggests that the early α-syn accumulation and LB formation is not necessarily induced by iron. The precise role of LB in the process of neurodegeneration is still unclear (Jellinger 2014), since its patho-mechanisms are unknown. Therefore, they may represent a cause or a consequence of cell death. In view of their early appearance in the asymptomatic phase of the illness (incidental Lewy body) (Gibb and Lees 1988a) coupled with their appearance in close vicinity in brain area exhibiting neuronal cell carnage, it, therefore, appears highly that α-syn plays some ominous role in neuronal destruction and progression. Indeed, in vitro and in vivo studies, suggest that α-syn undergoes a conformational change to a toxic form and thus initiating or subscribing to neuronal cell destruction (Lashuel et al. 2013).

Based on its location in the pre-synaptic area, its has, therefore, been suggested that the physiological role of α-syn may be in the release of neurotransmitters (Sulzer and Edwards 2019). Whereas, over expression of α-syn, appears to block the neurotransmitter release from the synaptic vesicles. Additionally, it is also involved in the regulation of mitochondrial fusion–fission (Bernal-Conde et al. 2020). It appears that α-syn is able to conduct physiological functions in the monomeric form. However, misfolding of the protein results in the conformational change to the toxic version of α-syn (this includes both the phosphorylated and non-phosphorylated form) and the formation of oligomers, which induces mitochondrial malfunction and ultimately triggering patho-mechanisms characteristic to the disease.

Factors such as ageing, genetic predisposition, selective vulnerability of areas in the brain, may contribute to the cellular degeneration capacity of α-syn (Wong and Krainc 2017). Indeed, mutations in the α-syn/SNCA gene (such as, A30P and A53T) augment the propensity for the α-syn to produce protofibrillar intermediates of its toxic oligomers (Bengoa-Vergniory et al. 2017). Perhaps these factors exert an indirect role, such that due to normal ageing coupled with some underlying genetic contribution the cellular protein homeostasis is disrupted, resulting in the accumulation of α-syn aggregates and thus the formation of LB. Furthermore, the interactions between α-syn and other PD proteins such as ubiquitin ligases encoded by PARK2 and PARK7 genes (Hauser et al. 2017) and kinase proteins, LRKK2 (Harvey and Outeiro 2018), suggest that α-syn may execute its pathological role via interaction with other proteins. This notion is also supported by its association with proteins involved in cellular destruction, such as PARK2, which regulates apoptosis and STUB1 that manages cell death (Kalia et al. 2011). Although, familial PD comprises for less than 10% of the cases, of which not many are related to SNCA mutations, thereby suggesting that the involvement of other factors, thus a collection of conditions, e.g. molecules coupled with an underlying genetic link may all represent key members of the pathogenic orchestra.

Interestingly, there appear to be different forms of α-syn protein congregations, which in turn give rise to various synucleinopathies including, PD, MSA and dementia with LB (Peelaerts et al. 2018). Thus, the unique misfolded α-syn conformational variants confer to the distinct type of synucleinopathies exhibited. This may be related to the differential accumulation processes and subsequent production of the α-syn variants. Additionally, this may also ascribe to the diverse clinical features of these neurodegenerative disorders.

### Neuroinflammatory aspects of Parkinson disease

α-syn aggregates have also been implicated in the activation of a chronic state of inflammation in PD (Hirsch 1994; Varanita and Bubacco 2020; Hirsch and Standaert 2021). In fact, the presence of active microgliosis in SN in PD was first suggested in the occurrence of neuroinflammation in the diseases process (McGeer et al. 1988). The microglia under physiological conditions exert a protective function on the brain via neurotrophic secretion. However, in the diseased state they adopt a pathogenic role. Therefore, perhaps a release of NM together with NM-bound components contributing to initiate and worsen an eventual immune response and exacerbating OS and neuroinflammation contributes to the cell death and progression of the disease. Possibly in the early stages of the illness, the nigral cell death may evoke inflammation as a neuroprotective response; however, as the disease progresses, the dying neurons may release inflammatory molecules that contribute to a state of chronic inflammation and consequentially exacerbating the cell destruction. However, it is unclear whether inflammation is primary or secondary to the neurodegeneration (Hirsch and Standaert 2021; Depboylu et al. 2007). It is very likely that there are other factors in addition to neuroinflammation that contribute to the cell death downstream (Halliwell 2006). Indeed, the brain is modestly equipped with anti-oxidant artillery and thus easily assailable and susceptible to get overwhelmed by aggressive degenerative processes such as inflammation, OS, mitochondrial dysfunction and inadequacy of the protein clearance system to destroy unwanted protein aggregates (such as misfolded α-syn oligomers).

### Glutathione–iron relationship

A plethora of evidence is indicative of the occurrence of oxidative stress in PD (Götz et al. 1990; Trist et al. 2019), although it is unclear whether it represents a cause or a consequence of the characteristic neurodegeneration observed in the SN. Nevertheless, its cytotoxic pathways and their products carry the lethal potential to evoke cellular destruction. OS is implicated by a host of nigral changes such as, elevation of total iron, increase in monoamine oxidase (MAO A) activity (Tong et al. 2017), changes of aldehyde dehydroxygenase (ALDH) activity (Mandel et al. 2005; Michel et al. 2014), loss of NM, an increase in lipid peroxidation, reduction in the antioxidant glutathione (GSH). It employs ROS or free radicals as leading culprits that interfere with physiological function causing cellular havoc and eventually neuronal destruction in its wake. It has been suggested that the dopamine degradation mechanisms may contribute to the production of ROS. Indeed, the elevation of dopamine metabolic enzyme, MAO A activity and loss of ALDH-1A supports this notion and in addition oxidation of dopamine produces toxic quinones and free radicals. Interestingly, the isoenzyme MAO B activity is increased in the frontal cortex but not in the SN (Tong et al. 2017). The main mode of free radical destruction is, antioxidants. However, no changes were reported in the antioxidant enzymes, total superoxide dismutase (Saggu et al. 1989) and catalase (Jenner et al. 1992a, b). Similarly, there was an absence of the change of activity of glutathione peroxidase (Sian et al. 1994), the enzyme responsible for the conversion of GSH to GSSG, thereby suggesting that the reduction of nigral GSH content (Sian 1991) is not due to its conversion to the oxidised form (GSSG), furthermore the GSSG content was unaltered in PD (Sian 1991). Interestingly, a depletion of glutathione appeared to evoke destruction of dopaminergic nigral neurons and protein aggregation in rats (Garrido et al. 2011). GSH is synthesized in both the neurons and the astroglial cells (Dringen and Hirrlinger 2003), although the glia has higher GSH levels compared to neurons since it is able to utilise a larger variety of substrates (Smeyne and Smeyne 2013). Indeed, studies using mercury orange for staining GSH showed reduction of GSH in the surviving neurons in contrast to the microglial cells in PD (Pearce et al. 1997). Perhaps activated glial cells are unable to provide cysteine required for neuronal GSH synthesis, thereby contributing to depletion of the GSH content in the neurons. Therefore, the neurons may be more susceptible to OS. In addition, the cytotoxic molecules released from microgliosis may exacerbate the neuronal damage. This suggests a significant role played by GSH in the neurodegeneration, as demonstrated by its reduction in the early asymptomatic phase of the illness, incidental Lewy body disease (Dexter et al. 1994). The early stages of the illness are marked by a triad of pathological changes including nigral cell loss (~ 40%), the presence of LB and loss of GSH. Perhaps the GSH serves as a triage and is “consumed” by early cell loss, subsequently the loss of cellular protection of the antioxidant may render the neurons vulnerable to the protein accumulation and cytotoxic free radical mechanisms and OS. Furthermore, in the SN symptomatic phase of the disorder there are more LB inclusions, a greater loss of SN neurons (~ 82%, (Iacono et al. 2015)), similar loss in GSH (Dexter et al. 1994), elevation in iron (Riederer et al. 1989) and a reduction in mitochondrial complex 1 (Schapira et al. 1990, 1989). Interestingly, the absence of any remarkable change in GSH loss in the symptomatic phase of PD compared to the asymptomatic phase, is bewildering, particularly in view of the escalation of neuronal cell death. Studies using human neuroblastoma cells suggest that an over expression of α-syn appears to alter the antioxidant capacity of GSH-deficient cells (Perfeito et al. 2017). Extrapolation of these findings would suggest, that possibly due to the progressively increasing formation of α-syn containing LB coupled with a depletion of GSH in the SN, this somehow compromises antioxidant ability of glutathione, thus implicating α-syn oligomers as a potential candidate for initiating neurodegeneration. It has been reported, that the antioxidant GSH is able to form complexes with iron such as FeS-glutaredoxins, that exert an important role in iron metabolism and trafficking (Berndt and Lillig 2017). Therefore, the depletion of nigral GSH in the early stages of the illness (Dexter et al. 1994) may at least in part contribute to the iron dyshomeostasis and subsequent elevation of iron in PD, as discussed above.

### Ferritin

Additionally, since both an increased (Riederer et al. 1988) and a decreased (Dexter et al. 1991) ferritin content in SN in PD have been reported, it is difficult to make any clear deductions. L-ferritin is part of NM granules (Tribl et al. 2009). This finding clearly proves NM/-L-ferritin as exclusive iron storing structure in dopaminergic neurons of the SN. Ferritin is the major iron-binding protein in glial tissue. However, the role of L- and H-ferritin, which are differentially distributed (Connor et al. 1994) and regulated on a post-transcriptional level has not been clarified in detail in human post-mortem tissue (Sammarco et al. 2008). A substantial decrease of L-ferritin concentration has been found in the SN by Connor et al. (1994), Connor and Menzies (1995) and Galazka-Friedman et al. (2004) even in ILBD. In contrast H-ferritin was higher in ILBD and controls (Koziorowski et al. 2007). As the ratio H/L-ferritin is substantially favouring H-ferritin, the different expression of both subtypes in the SN of PD deserves further attention, especially as the L-chain isoform is associated with iron long-term storage and the H-chain isoform predominates high iron-turnover (Boyd et al. 1985), suggesting a different mode of iron handling in the brain. Additionally, the loss of iron chelator, NM, may also be associated with the elevation of nigral iron levels in PD. Consequently, dyshomeostasis of the Fe(III):Fe (II) ratio develops (Sofic et al. 1988), which is another pathognomonic of the disorder. It should be mentioned that Sofic et al. (1988) were interested to measure the total amount of iron in the SN, i.e. free/labile iron, iron bound to neuromelanin and ferritin as well as iron bound to tyrosine hydroxylase or other proteins. Therefore, tissue was pretreated with hydrochloric acid and pepsin. Fe(III) executes cellular destruction via triggering cytotoxic mechanisms (Minotti and Aust 1987). Indeed, the elevated iron promotes the Fenton and Haber–Weiss reaction and can exacerbate the production of free radicals such as toxic hydroxyl radical species from hydrogen peroxide (Youdim et al. 1993). These reactive species initiate processes such as cellular lipid peroxidation, mitochondrial dysfunction, cell blebbing and eventually destruction of the neuron.

### Mitochondrial dysfunction

Indeed, free radicals can produce damage to mitochondrial DNA resulting in its loss of function (Liu and Chen 2017). Similarly, loss of mitochondrial function can also be produced by mutations in mitochondrial DNA as observed in familial PD. Consequently, disruption of mitochondria function may provoke disastrous events such as reduction of respiratory chain activity reported in PD (Schapira et al. 1989, 1990; Reichmann and Riederer 1989; Mizuno et al. 1989), production of more free radicals, profound effects on iron metabolism since mitochondria is involved in the production of haem and iron cluster proteins (Richardson et al. 2010), thereby suggesting that the nigral mitochondrial dysfunction may affect the iron metabolism via activity of ferrireductase/α-syn and subsequently resulting in the elevation of iron in PD. It appears highly likely that the pathological changes in biochemical parameters are closely associated and can prompt the initiation of cellular deleterious mechanisms. Mitochondrial dysfunction generates OS, reduced synthesis of iron–sulphur clusters and activation of iron regulatory protein 1. By this, accumulation of iron occurs, which causes hydroxyl radical-mediated damage (Muñoz et al. 2016). Additionally, ageing and underlying genetic predisposition are key players that orchestrate pathways. In fact, it has been suggested that α-syn accumulations’ and ageing can advocate a reduction in mitochondrial sirtuin 3, which physiologically plays a vital role in mitochondrial function and protection against OS (Park et al. 2020). Misfolded α-syn aggregates have the potential to invoke many of the pathological changes reported in PD, thereby endorsing its importance in the pathogenesis of the disorder.

The appearance of these α-syn oligomers also suggest a failing protein clearance system, namely the ubiquitin–proteasome pathway. This concept is supported by the presence of p62 in the ubiquitinate aggregate of LB in PD (Zatloukal et al. 2002). Malfunction of p62 would ascribe to the build-up of unnecessary α-syn aggregates and LB formation since it executes the breakdown of incorrectly folded proteins. Furthermore, since it regulates protein homeostasis via ubiquitin–proteasome system and autophagy, this therefore highlights p62 as a focal point in the faulty protein clearance system in the pathology of the illness (Shin et al. 2020). Interestingly, it was reported that rotenone, an inhibitor of the respiratory chain activity, produced overexpression of p62 which was associated with α-syn aggregates (Wu et al. 2015). Extrapolation of these finding would suggest a genetic component that induces the overexpression of p62 and the α-syn accumulation and LB formation.

### Bacteria– and SARS-CoV-2–iron interaction

Braak suggested that PD pathology may begin in the periphery spreading to the glossopharyngeal and vagal nerve and to the brain (Braak et al. 2003a). Possibly, micro-bacteria residing in the gut may produce inflammation resulting in aggregation of α-syn. Indeed, an imbalance of gut bacteria or a state of dysbiosis may act as pro-inflammatory mediators (Sherer et al. 2003; Dodiya et al. 2020). Mice treated with the complex I inhibitor, rotenone, exhibited (1) intestinal hyperpermeability, (2) glial cells inflammation, (3) increase in α-syn levels, (4) an increase of Gram-negative bacteria and (5) OS (Dodiya et al. 2020). Interestingly, there was also a reduction of “anti-inflammatory” bacteria such, *Lactobacillus*. This protective role may suggest that the bacteria is iron-independent similar to *Lactobacillus plantarum* (Archibald and Fridovich 1983), although iron is closely associated to most gut microbiota since it is vital for the propagation and survival of bacteria. Furthermore, it appears that iron may execute a rather ambivalent role, since low dietary iron appeared to produce dysbiosis of gut bacteria (Dostal et al. 2013). Conversely, an iron rich environment appears to support the growth of pathogenic bacteria such as *Proteobacteria* and induce inflammation (Jaeggi et al. 2015; Xiang et al. 2020).

Nevertheless, an association between iron, specific gut bacteria and inflammation is evident. It is likely that in PD the gut iron levels are elevated and this may produce OS, a state of dysbiosis and reproduction of bacteria that induce α-syn modification and inflammation. The inflammation may alter the environmental pH which may induce the α-syn misfolding and aggregation (Meade et al. 2019; Fitzgerald et al. 2019). Subsequently, these α-syn accumulations may then be transported and deposited from gut via the vagus nerve to the brain in a retrograde and time-dependent manner. Indeed, α-syn aggregates from PD brain lysate injected into the submucosa of the enteric nervous system of mice endorsed this notion (Holmqvist et al. 2014; Fitzgerald et al. 2019). The dual-hit hypothesis suggests that a potential pathogen may enter the brain through the gut and the olfactory system and a two-way communication exists between the microbiota in the periphery and central nervous system (Perez-Pardo et al. 2017). Additionally, a disturbed microbiota gut-brain axis may advocate a pathogenic role via triggering inflammation, α-syn oligomerisation and OS in PD. Interestingly, iron homeostasis and microbiota are said to be significant in eliciting intestinal inflammation (Yilmaz and Li 2018). Therefore, the notion is of interest, that even viruses selectively infect iron-acquiring cells or interact with cellular iron metabolism, including hepcidin cells (Schmidt 2020). Hepcidin is a central regulator of systemic iron homeostasis (Nemeth and Ganz 2009). SARS-CoV-2 interacts with haemoglobin through CD 147, CD 26 and other receptors on erythrocyte and/or blood cell precursors and via hepcidin-mimetic action of a viral spike protein, including ferroportin blockade (Cavezzi et al. 2020). Furthermore, SARS-CoV-2-induced COVID-19 manifests in inflammation, immune dysfunction and hyperferritinemia suggesting iron overload (Habib et al. 2021). Gastrointestinal symptoms are frequent in patients with COVID-19 (Villapol 2020; Huang et al. 2021; Weng et al. 2021; Buscarini et al. 2020; Xiang et al. 2020; Riederer and Meulen 2020; Vetter et al. 2020). For long time, iron-binding proteins like transferrin, lactoferrin and ferritin have been associated with cells of the immune system, and regulatory effects of metals on immune cells, including iron, have been suggested to cause toxic effects (Brock and de Sousa 1986). SARS-CoV-2 binds to ACE-2 and the type-II transmembrane serine proteases TMPRSS2 at the body’s epithelial cells. Furthermore, TMPRSS11D and TMPRSS13 activate the SARS-CoV-2 spike protein (Kishimoto et al. 2021). Other type-2-transmembrane serine proteases have not been studied so far on the replication of SARS-CoV-2. TMPRSS 6 gene is related to the generation of the protein matriptase-2 (Ramsay et al. 2009), which controls hepcidin biosynthesis and by this iron homeostasis. Indeed, a distant sequence similarity between the cysteine-rich cytoplasmic tail of the coronavirus spike protein and the hepcidin protein has been described recently (Ehsani 2020). Therefore, it is not farfetched to assume, that iron homeostasis is disturbed in various organs due to viral infection and thus contributes to the pathology induced by SARS-CoV-2. Indeed, Nai et al. (2021) in a clinical study involving 111 COVID -19 patients showed that serum iron was extremely low in most cases and that this finding was a predictor of mortality. Conversely, hepcidin levels were significantly increased in 61.3% of patients. Patients with higher hepcidin levels were significantly older and had higher concentrations of markers of inflammation, i.e. CRP and ferritin and cell damage, like LDH. Low serum levels of iron and increased concentrations of both ferritin and hepcidin are characteristic markers of patients with COVID-19 infection (Sonnweber et al. 2020; Hippchen et al. 2020; Cavezzi et al. 2020; Cheng et al. 2020; Henry et al. 2020; Nai et al. 2021; Girelli et al. 2021; Mahat et al. 2021).

In addition, iron deficiency was still present 60 days after disease onset in 30% of subjects. Anaemic patients (9%) had increased markers of inflammation (IL-6, C-reactive protein). Hyperferritinemia was still present in 38% of all individuals and was more frequent in severe or critical COVID-19 (Sonnweber et al. 2020). Hepcidin is rapidly and potently stimulated by pro-inflammatory cytokines like IL-6, leading to hypoferremia, impaired haemoglobin synthesis causing anaemia or inflammation (Girelli et al. 2021; Nai et al. 2021).

As SARS-CoV-2 seems to have hepcidin-like action, the virus can directly increase ferritin levels. Furthermore, the release of iron may be secondary to the interaction between SARS-CoV-2 and haemoglobin and this may cause high ferritin concentration (Garrick and Ghio 2021; Abobaker 2021). Indirect evidence of altered iron homeostasis comes from clinical studies in optimal individualized therapy plus deep brain stimulated patients with PD. Here blood levels of hepcidin and IL-6 concentrations in blood were significantly elevated, indicating neuroinflammatory induced disturbances of peripheral iron homeostasis (Kwiatek-Majkusiak et al. 2020). It is suggested, that more attention should be given to the interaction of iron with SARS-CoV-2 and other viruses. Such studies may contribute to understand the viral toxicity on organs functions. In the 6-hydroxydopamine- as well as in the rotenone-induced PD models overexpression of hepcidin suppressed major pathologies of parkinsonism, protected rotenone-induced mitochondrial deficits and reduced α-syn accumulation through a decrease of iron (Liang et al. 2020). Interleukin-6, which is significantly increased in the SN of PD (Mogi et al. 1994) and chronic inflammation (Hirsch and Standaert 2021) increases hepcidin levels (Camaschella et al. 2020). Therefore, the interplay of hepcidin, iron-transport proteins like transferrin, lactoferrin and ferroportin and iron-storing ferritin as well as NM controlling the concentration of free as well as bound/stored iron is a critical factor in the pathology of PD (Vila 2019). Therefore, chelation of excess peripheral free iron as well as excess of SN free/labile iron with iron chelators, which cross the BBB only at the site of SN BBB disturbance, we suggest to be potential targets for new drug developments to causally influence the iron-induced pathology of PD.
